# The Student's Bitter Cry

**Published:** 1888-09-15

**Authors:** 


### THE STUDENT'S BITTER CRY.

The medical student, when all has been said, is really the person most concerned in the character and doings of the medical schools. It is he who has to do the prescribed work, pass the prescribed examinations, and, finally, to use his knowledge and skill which the schools have taught him all the rest of his days for a livelihood. Therefore, though parents are profoundly anxious and deans deeply interested, the student, as being the real "bone of contention," may be permitted to have a word. The briefer and plainer that word is the better. The student enters the medical school on a very different level from that of the youth who goes to college to obtain a degree in arts. The arts student merely continues the Latin, Greek, mathematics, and so on at which he has been working during the whole of his school life. He has therefore a good elementary groundwork and a perfect familiarity with the methods of study in general use. But the youthful medical student so soon as he has passed his preliminary examination enters upon studies which in nine cases out of ten are as new to him as shipbuilding, or navigation, or ballooning would be. Nobody seems to be aware of this except the student himself, and he is grievously aware of it. He finds chemistry, as it is usually taught, most difficult to learn, whilst the reading of the bones is hideous. Why is he not taught? That is the question which he wants answered. Take chemistry as an example. It is one of the most interesting and easily learnt of subjects when it is taught experimentally. In three months a young man in a laboratory, with an enthusiastic teacher, could learn more than most medical men ever know during their whole lives. But how many medical students ever see the inside of a laboratory? It is at bottom a question of expense and trouble. Much the same might be said of physiology and pathology. They are not taught; they are lectured about. Lecturing, as a supplement to teaching, is admirable when carried out by competent men; but when it is used, not as a supplement, but as a substitute, it is a complete failure. Medical and scientific teachers at hospital medical schools are not appointed because they can teach, but because they desire hospital appointments to help them in practice, and because they have influence enough to get them. This is an absurdity and a scandal, of which any doctor who vaunts the necessity and value of sound training ought to be ashamed. If a man reads the plausible articles which appear in newspapers, and takes his cue from them, he will be under the impression that in hospitals and the medical profession generally everything is subservient to the general advancement of science, and to the special object of making each medical student a highly-trained and scientific practitioner. What a blinded and deceived fool such a man would prove himself to have been if he were to spend two years at an ordinary medical school. No man, whatever his knowledge, should be appointed teacher at a medical school unless he can teach, or lecturer unless he can lecture. That is the first reform to be made. The second is that there should be at least six times as many teachers employed as there now are. Then students would be taught in detail. The very best medical students, who take honours and gold medals, seldom or never know their work practically and intelligently as they ought to do. They are excellent crammers, and, very naturally, they pride themselves on their successes. But of real practical intelligence and detailed skill many of them have little or none. The system of teaching at many medical schools is as bad as bad can be, and is a disgrace to the science of the times.

